# Medical Image Segmentation Using Fruit Fly Optimization and Density Peaks Clustering

**DOI:** 10.1155/2018/3052852

**Published:** 2018-12-24

**Authors:** Hong Zhu, Hanzhi He, Jinhui Xu, Qianhao Fang, Wei Wang

**Affiliations:** ^1^School of Medical Information, Xuzhou Medical University, Xuzhou, China; ^2^Key Laboratory of Intelligent Industrial Control Technology of Jiangsu Province, College of Information and Electrical Engineering, Xuzhou University of Technology, Xuzhou, China; ^3^Department of Computer Science and Engineering, State University of New York at Buffalo, Buffalo, USA; ^4^Department of Medical Imaging, The Affiliated Hospital of Xuzhou Medical University, Xuzhou, China

## Abstract

In this paper, we propose a novel algorithm for medical image segmentation, which combines the density peaks clustering (DPC) with the fruit fly optimization algorithm, and it has the following advantages. Firstly, it avoids the problem of DPC that needs to artificially select parameters (such as the number of clusters) in its decision graph and thus can automatically determine their values. Secondly, our algorithm uses random step size, instead of the fixed step size as in the fruit fly optimization algorithm, which helps avoid falling into local optima. Thirdly, our algorithm selects the cut-off distance and the cluster centers using the image entropy value and can better capture the structures of the image. Experiments on benchmark dataset and proprietary dataset show that our algorithm can adaptively segment medical images with faster convergence and better robustness.

## 1. Introduction

Segmentation is a key step in medical image analysis. It helps avoid the interference from the area outside of the region-of-interest (ROI) and allows a more accurate extraction of the features (such as the shape, texture, etc.) of the diseased tissues. Thus, it is of great significance for disease prediction and adjuvant therapy for the lesion [1–3].

With the rapidly advancing technologies in medical imaging, more and more medical procedures are now heavily relying on medical images. For this reason, massive volume of medical images is generated daily. This imposes a great challenge to image analysis. Manual segmentation is obviously time consuming and inefficient and thus cannot meet the demands of high throughput extraction of the big medical image data. Therefore, developing fully automatic algorithms for efficiently and accurately segmenting medical images is becoming a big and urgent issue in medicine. Due to its importance, extensive research has done on this problem and a number of approaches have been proposed, such as threshold methods, clustering algorithms, entropy-based segmentations, artificial neural networks, region growing methods, etc. Among all these approaches, deep learning based methods have gained a lot of popularity in recent years, due to their high quality of segmentations. However, such methods often require abundant samples as the training data [4, 5], which may not always be available for some types of medical images. Thus, clustering-based segmentation algorithms, such as K-means [6–8], fuzzy C-means (FCM) [9–12], and density-based clustering [13–15], are still good alternatives, due to their unsupervised nature. Researchers have conducted in-depth research on image segmentation and proposed various effective methods. Reference [16] proposed a computerized tool based on the integration of Tsallis entropy and the seed region growing approach. It provides better results for brain MRI. A novel real time integrated method is developed in literature [17] based on the region growing segmentation method along with the thresholding supported image segmentation. In literature [18], rough-set theory can be a useful method to overcome complications during image segmentation. The results prove that the proposed method outperforms the region growing method in terms of the recall and F-score. For the improved methods proposed by these researchers, there are a lot of distance calculations, and the clustering problem with a large amount of data will result in a very high spatial complexity, which cannot be effectively dealt with for complex medical images.

Traditional clustering methods have mainly focused on the relationships among neighboring data points (e.g., pixels or voxels). Recent clustering algorithms have considered the relationships between any pair of data points and demonstrated better quality of solutions, due to their ability of utilizing the global information of the underlying structures.

One of such techniques is the affinity propagation (AP) [19] clustering algorithm, which was introduced in 2007 to simultaneously consider all data points as potential exemplars. By viewing each data point as a node in a network, it recursively transmits real-valued messages along edges of the network until a good set of exemplars and corresponding clusters emerge. In this way, AP can overcome some drawbacks of K-means and fuzzy c-means and be applied widely in medical image segmentation [20–22]. AP clustering method uses the Euclidean distance to measure the similarity and ignores the shape information of the region-of-interest. However, due to the complexity of human anatomy and the irregular shapes of human tissues and organs, Euclidean distance is often not sufficient to fully capture the similarity. Thus, better algorithms are still needed for medical image segmentation.

Another such technique is the density peaks clustering (DPC) [23] method, which is based on the idea that cluster centers have higher density than their neighbors and relatively large distance from points with higher densities. It considers all data points as candidate clustering centers. For each data point, DPC computes its local density and its distance from points of higher density. In this way, it utilizes the global information of the data. Compared with similar algorithms such as the AP method, it can find arbitrarily shaped clusters and outliers automatically. In addition, it does not require embedding the data in a vector space like mean-shift, and it needs not select the seed blindly like DBSCAN. DPC is simple and efficient as it uses only the distances between data points. It is suitable for medical image processing [11, 24, 25], but it is not easy to select the proper parameters.

The metaheuristic algorithms are good solutions to optimization problems. They mainly include genetic algorithm, ant colony optimization algorithm, particle swarm optimization algorithm, and so on. Several researchers have done research. References [26, 27] proposed a new approach of Cuckoo Search (CS) to select the optimal threshold value. MSE and PSNR are measured to understand the segmentation quality. References [28, 29] discussed several medical applications using metaheuristic-based approaches for segmentation, and a novel approach to deal with rats microscopic hippocampus images segmentation based on the hybrid evolutionary strategy (ES) is proposed. The results have superior segmentation with eight levels. References [30] proposed the ant weight lifting (AWL) which is inspired from the behavioral nature of ants. It adds perk in the form of a low time complexity. These optimization algorithms also have their own advantages and disadvantages. The cuckoo algorithm and the ant colony algorithm are computationally intensive, too complex, and easy to prematurely converge. In contrast, the fruit fly optimization algorithm is a relatively novel efficient metaheuristic algorithm proposed in recent years. The algorithm is simple to implement and the calculation amount is small.

To resolve the aforementioned issues with DPC, we present in this paper an improved DPC algorithm based on the fruit fly optimization and apply it to medical image segmentation. The algorithm is a judicious combination of the fruit fly optimization algorithm and the density peaks clustering and can resolve some defects in DPC algorithm, such as the cut-off distance *d*_c_ was given by DPC algorithm relied on prior knowledge and subjective randomness in cluster centers was selected by manual work. We change the fixed step size to random step size in the fruit fly optimization algorithm, which helps avoid falling into local optima. In addition, our algorithm selects the cut-off distance and the cluster centers using the image entropy value and can better capture the structures of the image. Experimental studies on benchmark medical image dataset and proprietary dataset show that our proposed algorithm outperforms existing methods.

The rest of this paper is organized as follows. In Section 2, we describe some fundamental concepts.  Section 3 presents the DPC algorithm for medical image segmentation, but the effect is not ideal. In Section 4, we describe the parameter selection for optimizing DPC algorithm using fruit fly optimization algorithm in more detail. The experimental results and discussion of these results on both public dataset and proprietary dataset are described in Section 5. Finally, we present the conclusion and the future work in Section 6.

## 2. Preliminaries

### 2.1. Density Peaks Clustering Algorithm

The cluster centers of DPC [11, 23–25] are points whose local densities are as large as possible and have large relative distances between other points with higher density.

For clustering dataset *S*={*χ*_*i*_}_*i*=1_^*N*^, (*N* ∈ *N*+), density peaks clustering algorithm defines local density *ρ*_*i*_ and relative distance *δ*_*i*_ for each data point *χ*_*i*_ in the data set *S*. These two variables are related to the distance *d*_*ij*_ between any two objects in the dataset.

The local density of data point *χ*_*i*_ is defined as

(1) $$\begin{array}{c} \rho_{i}=\underset{j}{\sum }\chi \left(d_{ij}-d_{\text{c}}\right), \end{array}$$

where

(2) $$\begin{array}{c} \chi \left(x\right)=\left\{\begin{array}{c} 1,\;x<0,\\ 0,\;x\geq 0. \end{array}\right. \end{array}$$

The parameter *d*_c_ > 0 is the region-of-interest distance which needs to be specified in advance. *d*_*ij*_ is the distance between data points *i* and *j*. Based on the above analysis, we know that *ρ*_*i*_ is the number of data points which is within the *d*_c_ range around the data point *i*.

The distance *δ*_*i*_ can be defined as

(3) $$\begin{array}{c} \delta_{i}=\left\{\begin{array}{cc} \underset{j:\rho_{i}>\rho_{j}}{\min}\left(d_{ij}\right), & i\geq 2,\\ \underset{j\geq 2}{\min}\left(d_{ij}\right), & i=1, \end{array}\right. \end{array}$$

where *δ*_*i*_ is the distance between data points *i* and the data points *j* which is the closest point to data point *i* among all points with a greater density than data point *i*. If a data point *i* has both higher *ρ*_*i*_ and larger *δ*_*i*_, it is more likely to be a cluster center. The method in [23] first uses qualitative analysis, that is, using the distribution of *ρ*_*i*_ and *δ*_*i*_ in the decision graph, to select the cluster centers manually, then classifies the remaining data points to the nearest clusters according to density from the largest to the smallest, and eventually obtains the clustering results.

The specific process of the DPC algorithm (Algorithm 1) can be described as follows:

Data points *i* and *j* are any points in data set *S*, *j* is the point closest to *i* in all points with higher density than data point *i*.

### 2.2. Fruit Fly Optimization Algorithm

Swarm-intelligent algorithm is one common methodology for optimizing the parameters of clustering method [31–33]. The fruit fly optimization algorithm (FOA) [34] is a new swarm-intelligent optimization algorithm proposed by Dr. Pan W T in 2012 and has been used widely in many fields [35‐37]. Fruit fly population has a strong sense of smell and vision. When a fruit fly smells a distant food, it flies towards the food source and sends or receives the position information of the food to or from its companions. After a number of smell-based search processes, the fruit fly performs a visual search to select the best odor concentration information and then flies to that location.

The fruit fly optimization algorithm can be divided into the following steps:

***Step 1*.**

Initialization:

Initializes the population size *Sizepop* and the maximum number of iterations *Maxgen*. Selects the position *X*_*axis*, *Y*_*axis* of the fruit fly population randomly in the search space.

***Step 2*.**

Smell-based searching process

***Step 2.1*.**

Calculate the random direction and distance of the smell-based food seeking of every fruit fly.

(4) $$\begin{array}{c} \left\{\begin{array}{c} X_{i}=X\_axis+\text{random}\;\;\mathrm{value,}\\ Y_{i}=Y\_axis+\text{random}\;\;\mathrm{value.} \end{array}\right. \end{array}$$

***Step 2.2*.**

Calculate the distance (Dist_*i*_) between each fruit fly and the origin. Calculate the concentration value (*S*_*i*_) of the smell, which is the reciprocal of the distance:

(5) $$\begin{array}{c} {\text{Dist}}_{i}=\sqrt{X_{i}^{2}+Y_{i}^{2}}, \end{array}$$

(6) $$\begin{array}{c} S_{i}=\frac{1}{{\text{Dist}}_{i}}. \end{array}$$

***Step 2.3*.**

The dominant value (*S*_*i*_) of smell is brought into the fitness function to calculate the smell (Smell_*i*_) of the location of the fruit fly.

(7) $$\begin{array}{c} Smell_{i}=Fitness\left(S_{i}\right). \end{array}$$

***Step 2.4*.**

Find out the best dominant value of smell and corresponding optimal locations for contemporary fruit fly populations:

(8) $$\begin{array}{c} \left[\begin{array}{cc} bestSmell & bestIndex \end{array}\right]=\max\left(Smell_{i}\right). \end{array}$$

***Step 3*.**

Visual-based search process

The optimal dominant value of smell *bestSmell* and its coordinate position information are retained, and other individuals in the group fly to the position:

(9) $$\begin{array}{c} Smellbest=bestSmell, \end{array}$$

(10) $$\begin{array}{c} \left\{\begin{array}{c} X\_axis=X\left(bestIndex\right),\\ Y\_axis=Y\left(bestIndex\right). \end{array}\right. \end{array}$$

***Step 4*.**

Iterative optimization

Repeat step 2 through step 3 and retain the better value until the number of iterations *Maxgen* is reached.

## 3. Medical Image Segmentation Based on Density Peaks Clustering

**Figure 1:**
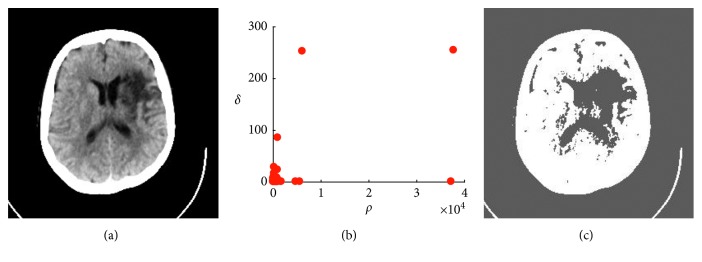
Manual selection of cluster centers and segmentation effects. (a) Cerebral stroke CT. (b) Decision graph. (c) Segmentation results.

DPC algorithm has been used for clustering data points since its invention. It can be applied to medical image segmentation in the following ways. Since most of the medical images are of high resolution, directly clustering them could be quite time consuming. Thus, our idea is to use DPC algorithm to cluster the gray values of all pixels. For each gray value, it defines the local density *ρ*_*i*_ and calculates its distance *δ*_*i*_ to other points with higher density. The medical images are preprocessed to extract gray value of the image. The abscissa of the gray histogram is the gray level, and the ordinate is the frequency of its appearance. In this case, the distance between each point is calculated, and the difference in gray level is used as the distance. We select the test data from Xray-CT images with Abnormal Tissue in Neuroimaging Primer in Harvard Whole Brain Database.  Figure 1(a) is a typical CT image of ischemic stroke. It can be seen that the lesion is the subcortical infarct of the left lateral ventricle and is accompanied by cortical edema of the middle cerebral artery, i.e., low-density lesion (dark) surround. We can draw a *ρ* − *δ* decision graph and manually select the points with larger *ρ*_*i*_ and *δ*_*i*_ values as the cluster centers (Figure 1(b)) and obtain the clustering results. The experimental results show that due to the complexity of medical images, unlike other types of data, the number of cluster centers that can be manually selected is small, and the qualitative analysis after segmentation is not very effective. Given the different cut-off distances *d*_c_ and the manually selected cluster center points, it is clear that the segmentation effect graph (Figure 1(c)) could not accurately reflect the lesions and the edema sites (i.e., undersegmentation). Many other experiments also show that the original DPC algorithm is sensitive to the selection of cluster center points.

## 4. Medical Image Segmentation Based on Fruit Fly Optimization and Density Peaks Clustering

The above experiments show that DPC algorithm cannot select the cut-off distance *d*_c_ adaptively in the process of medical image segmentation. It is also difficult to obtain good clustering results if we manually select the cluster center points. This motivates us to propose a new algorithm, called density peaks clustering based on fruit fly optimization algorithm (FOA-DPC), which can automatically select the DPC parameters according to the maximum entropy value of the medical image.

Our algorithm first calculates the local density *ρ*_*i*_ and its distance *δ*_*i*_ for each gray value and determines the clustering centers by these two parameters in the following way. First, another parameter *γ*_*i*_ can be defined as follows:

(11) $$\begin{array}{c} \gamma_{i}=\rho_{i}*\delta_{i}. \end{array}$$

**Figure 2:**
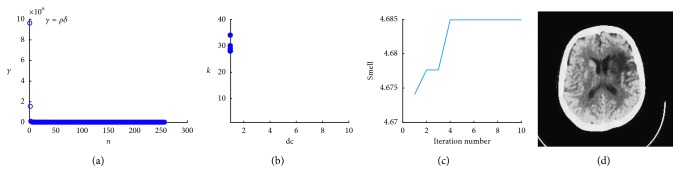
Decision of cluster center. (a) Descending order graph of *γ*. (b) Fruit fly optimization flying route. (c) Optimization process with iterations. (d) Effect of image segmentation.

The larger the *γ*_*i*_ value is, the more likely it is a clustering center. Thus, the *γ*_*i*_ values are then sorted in a descending order, and the first *k* points are taken as the clustering centers (as shown in Figure 2(a)).

Our algorithm then uses the cut-off distance *d*_c_ and the number of cluster centers *k* as the decision variables, which correspond to the *X*_*axis* and *Y*_*axis* in the fruit fly optimization algorithm. The key to iteratively optimizing the two parameters is to construct a smell concentration function (also known as fitness function) to screen the optimal solution in the offspring.

For this purpose, we first introduce the concept of entropy. Image entropy is a statistical form of features. It is an index of information entropy to measure the average information content of an image during the process of digital image processing. We use the one-dimensional entropy, which reflects the overall information of the image, to represent the amount of information contained in the gray value distribution of the image. The higher the image entropy, the clearer the image and the richer the content. The one-dimensional entropy of a gray image can be defined as

(12) $$\begin{array}{c} H=\overset{255}{\underset{i=0}{\sum }}p_{i}\;\;\log\;p_{i}, \end{array}$$

where *p*_*i*_ represents the probability that a pixel with a gray value of *i* appears in the medical image.

The gray value range of images is usually an integer from 0 to 255 in digital image processing. The domain of *d*_c_ should make the average number of data neighbors not more than two percent of the total according to [23]. Thus, the value of *d*_c_ should be within the range from 1 to 10. *k* represents the number of classifications that should not be less than 2 categories. According to the empirical studies of medical image segmentation, the number of categories should not be too large, and should have a value between 2 and 40.

The original fruit fly optimization algorithm uses a fixed step in both smell-based search process and visual-based search process, which can easily trap the algorithm into local optima and thus affects the convergence and stability of the algorithm. Since the DPC parameters to be optimized in this paper are not very large, a random step is used (for escaping the local optima) as a guide for the range of fly's activity. Random numbers are taken from the range between −5 and 5, and the defined variables are in the positive range.

To combine the fruit fly optimization algorithm with the DPC algorithm, our proposed algorithm uses image entropy, which reflects the overall information of the image, as the smell concentration function. It searches for the optimal segmentation threshold in the contemporary fruit fly population, as well as in the global search space, so that the segmented image entropy is maximized. Our algorithm calculates the optimal smell value and records the parameters corresponding to the optimal smell concentration for each generation as shown in Figure 2(b). A trend graph of the optimal smell concentration can be drawn (Figure 2(c)). The figure shows that after 5 iterations, the smell concentration basically converges. After multiple runs, we obtain the values of parameters *d*_c_, *k*, and the optimal fitness value as 1, 28, and 4.685, respectively. The segmentation results are shown in Figure 2(d). It can be seen that the segmented lesions and edema sites are clearly visible, which can help the doctor make the best judgments.

Our proposed segmentation algorithm (Algorithm 2) (based on the density peaks clustering algorithm and the fruit fly optimization algorithm) has the following main steps. The preprocessing of images includes reading medical images, extracting gray values, calculating image gray histograms, etc.

## 5. Experimental Results and Analysis

### 5.1. Experimental Design

#### 5.1.1. Experimental Environment and Dataset

The experimental hardware platform for this paper is Windows7 64-bit operating system, Intel Core i5-6500 CPU, 4 GB memory, and the algorithm is implemented in MATLAB-R2016 b environment.

Common brain diseases include brain tumors, traumatic brain injury, acute cerebrovascular disease, brain atrophy, etc., and their imaging features are different. Multiple MRI images of brain cases in Harvard Whole Brain Database were selected in the experiment, including T2-weighted images of stroke, meningioma, sarcoma, and metastatic bronchial carcinoma.

#### 5.1.2. Comparison of Algorithms

We compare our improved algorithm FOA-DPC with the original DPC algorithm, the classical algorithm K-means, and density peaks clustering (based on genetic algorithm) (GA-DPC), using both analytic and experimental methods, and investigate the effectiveness of our improved algorithm.

1. DPC algorithm: The tailored algorithm for medical image processing has been discussed in Section 2, which does not need to iterate.
2. K-means algorithm: It is the classical unsupervised learning algorithm which has been widely used in many fields.
3. GA-DPC algorithm: It is an image segmentation method based on improved density peak clustering which uses genetic algorithm to select the optimal parameters. It uses image entropy as the best fitness discriminant function to realize the unsupervised segmentation of images.

#### 5.1.3. Assessment Method

The variance between classes and image entropy can quantitatively measure the effectiveness of image segmentation. The larger the value is, the greater the difference between different classes and the richer image content is. Comparing the variance between classes is to judge the quality of the segmented image according to the size of the contrast between regions. The variance between classes is defined as follows:

(13) $$\begin{array}{c} \text{SEC}=\overset{k-1}{\underset{i=1}{\sum }}\left(\frac{N}{N+M}{\left(U_{1}-U\right)}^{2}+\frac{M}{N+M}{\left(U_{2}-U\right)}^{2}\right), \end{array}$$

where *k* is the number of cluster centers, *N* and *M* refer to the area of the first and second regions, respectively, which are generally the number of pixels in adjacent regions, *U*_1_, *U*_2_ are the average gray values of the first and second regions, respectively, and *U* is the average gray value of the two regions.

### 5.2. Algorithm Analysis and Experimental Results

#### 5.2.1. Algorithm Analysis

We compare the performance of FOA-DPC algorithm theoretically with K-means, DPC [23] and GA-DPC (discussed in 5.1.2) from several aspects, such as prior information, algorithm type, time complexity, robustness, etc. The result is listed below (Table 1).

Comparing to the K-means and DPC algorithms, we find that GA-DPC algorithm and FOA-DPC algorithm are both combined with some intelligent algorithms and thus do not need to specify beforehand the clustering number. Hence, they can be preceded with the advantage of autonomous segmentation of images in the absence of prior knowledge. In terms of time complexity, in Table 1, *n* is the number of data elements, *k* is the number of cluster centers, *t* is the number of iterations, and *p* is the population number. The DPC algorithm has the same magnitude as its improved algorithm, but higher than the K-means algorithm. The complexity of GA-DPC algorithm is similar to that of our improved algorithm. However, the genetic algorithm needs larger populations and more iterations and is thus more difficult to converge. The fruit fly optimization algorithm has a quicker convergence and shorter running time. This means that it has a higher search capability. In most cases, FOA-DPC chooses the most correct parameters in both public dataset and proprietary dataset, indicating that it is more robust. It will be explained in section 5.2.3.

#### 5.2.2. Experimental Results on Public Dataset

**Figure 3:**
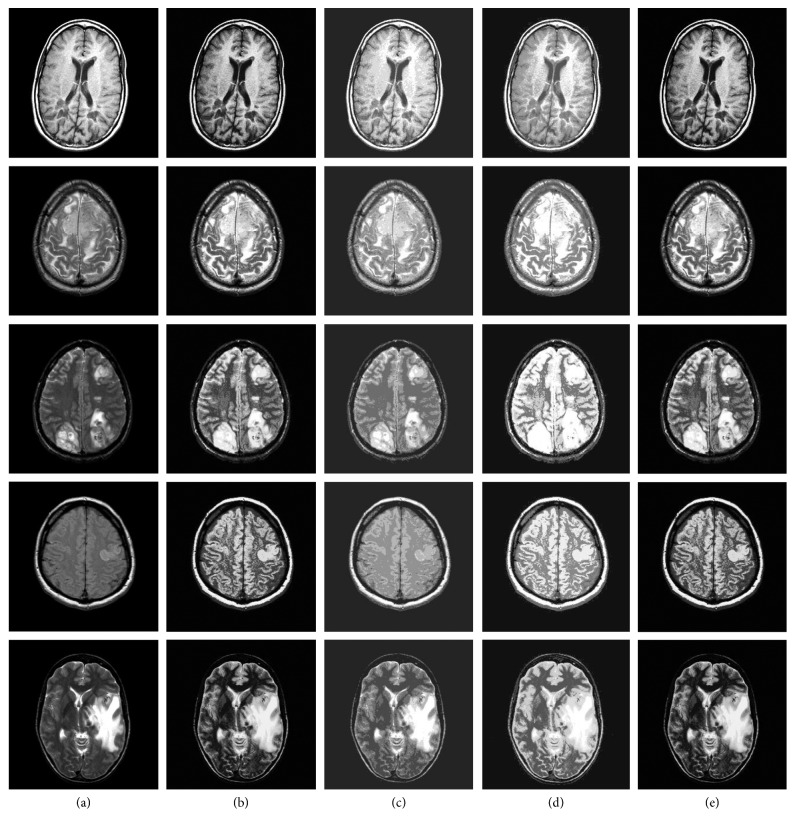
Segmentation results of four algorithms on brain MRI images. Column (a): original images; column (b): FOA-DPC; column (c): K-means; column (d): DPC; column (e): GA-DPC. Row 1: MS plaques; row 2: meningioma; row 3: sarcoma; row 4: acute cerebral infarction; row 5: metastatic bronchial cancer.

Our experiments use the public available MRI T2-weighted images of Harvard Whole Brain Database. For the K-means and DPC algorithms, experiments are performed using the original code provided by the authors. For each of the comparison algorithms, the internal parameters are set to their best values. For example, according to prior knowledge of medical images, when K-means algorithm parameter *k* is 7, the segmentation result is the best. The cut-off distance *d*_c_ and cluster number of the DPC algorithm *k* are, respectively, set to 3 and 15. Our FOA-DPC algorithm and the GA-DPC algorithm can adaptively select parameters. The experimental results are shown in Figure 3. It can be seen that our FOA-DPC algorithm and the GA-DPC have similar segmentation effect and are superior to the other algorithms.

Table 2 shows the average of multiple experiments. The information entropies of the medical images segmented by FOA-DPC algorithm and the GA-DPC algorithm are larger than those of the K-means and DPC algorithms. Compared with the GA-DPC algorithm, FOA-DPC algorithm always converges faster, although the result of optimization is consistent, and the overall computational time is about 1/4 of the GA-DPC algorithm. In contrast, compared with the simpler K-means and DPC algorithms, it takes more time which is mainly spent on the fruit fly iterative optimization. In general, the FOA-DPC algorithm has the advantages over other segmentation algorithms when the time consumption is acceptable. The experimental results are consistent with the theoretical analysis.

#### 5.2.3. Experimental Results on Proprietary Dataset

In this study, enhanced T1W1-weighted DICOM images of meningioma from a local hospital are selected. The enhanced meningioma showed significant and uniform hyperintensity, and the meningeal attachment of the meningioma was significantly enhanced by tumor infiltration. The cut-off distance *d*_c_ and cluster number of the DPC algorithm *k* are, respectively, set to 1 and 15. AP, FOA-DPC, and GA-DPC algorithms can adaptively select their own parameters. The GA-DPC population is 10, and the number of iterations is 20 to achieve convergence. Since the fruit fly optimization algorithm converges faster, we set the FOA-DPC population and the number of iterations both as 10.

**Figure 4:**
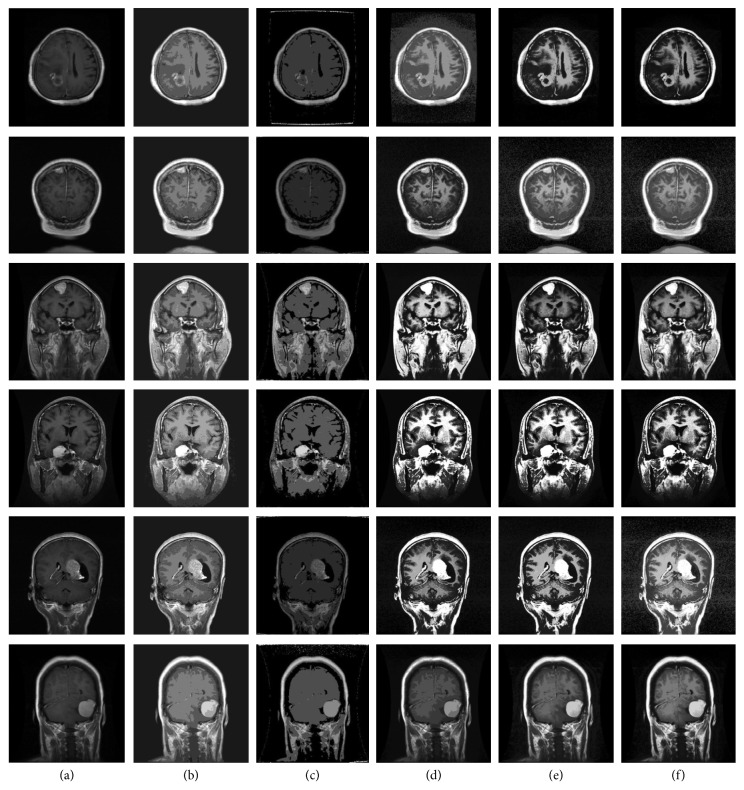
Segmentation results of four algorithms on proprietary dataset. (a) Original images. (b) K-means. (c) AP. (d) DPC. (e) GA-DPC. (f) FOA-DPC.

The experimental results are shown in Figure 4 and Table 3.

It can be seen from the Table 3 that FOA-DPC algorithm does not require prior knowledge for the complex medical images with no obvious regional gray scale difference and large number of clusters, but the segmentation effect is the best. FOA-DPC algorithm has higher image entropy than all other algorithms and can select its parameters adaptively, which leads to better segmentation results. FOA-DPC algorithm can always find the maximum value, which is the optimal parameter, so it has the characteristics of simplicity, high efficiency, and stronger robustness. The SEC values are also larger than the other algorithms basically, which shows that the FOA-DPC algorithms retain more information when segmenting gray scale images, and the segmented results are closer to the original images and the differences between different classes are greater. Thus, their segmentations are better.

## 6. Conclusions

In this paper, we proposed an improved algorithm FOA-DPC for medical image segmentation. It combines the density peaks clustering (DPC) algorithm with the fruit fly optimization algorithm, which uses image entropy as the best smell concentration discriminant function. The fixed step size of DPC has changed to a random step size to move the fruit fly, which largely avoids falling into local optima and is capable of adaptive segmentation of the image. Experiments on benchmark dataset and proprietary dataset showed that our FOA-DPC algorithm is effective and robust and can greatly reduce the segmentation time of the combinatorial swarm intelligence algorithm (such as GA-DPC). Despite its simplicity and high efficiency, our proposed algorithm still has some room for further improvements, such as how to reduce the iterative and computational complexities of swarm intelligence algorithms and how to apply it to the PET color image segmentation. We leave them as future work.

## Figures and Tables

**Algorithm 1:**
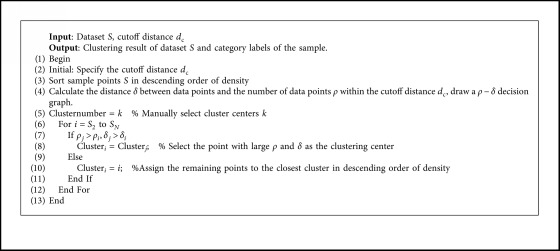
**DPC** Algorithm.

**Algorithm 2:**
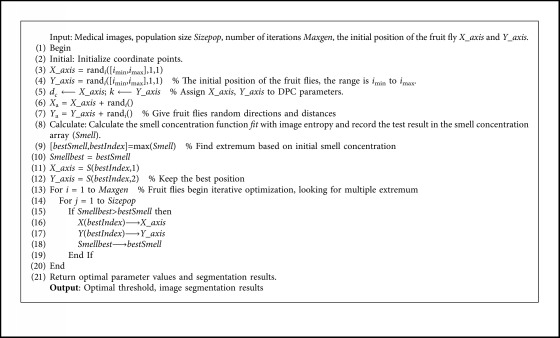
**FOA-DPC** Algorithm.

**Table 1:** The qualitative analysis of K-means, DPC, GA-DPC, and FOA-DPC algorithms.

Index of analysis	K-means	DPC	GA-DPC	FOA-DPC
Prior information	Specify cluster numbers in advance	Specify cluster numbers in advance	No need to specify cluster numbers in advance	No need to specify cluster numbers in advance
Algorithm type	Based on division	Based on density	Based on density	Based on density
Time complexity	O (*n* ∗ *k* ∗ *t*)	O (*n*^2^)	O (*p* ∗ *t* ∗ *n*^2^)	O (*p* ∗ *t* ∗ *n*^2^)
Robustness	Weak	Weak	Strong	Stronger

**Table 2:** Comparison of evaluation index values of K-means, DPC, GA-DPC, and FOA-DPC clustering algorithms.

Methods	Index	Experimental images				
		1	2	3	4	5
K-means	SEC	1793.24	1858.31	1426.70	1512.50	1549.43
	Image entropy	4.73	4.11	4.31	4.09	4.31
	Time/s	0.11	0.10	0.11	0.12	0.10

DPC	SEC	1701.49	1518.55	1590.32	1724.85	1793.05
	Image entropy	4.91	4.20	4.40	4.39	4.69
	Time/s	0.34	0.36	0.35	0.33	0.32

GA-DPC	SEC	1935.54	2188.63	2105.17	2105.01	1760.83
	Image entropy	5.04	4.39	4.72	4.50	4.77
	Time/s	40.21	39.99	40.18	40.25	40.14

FOA-DPC	SEC	1935.54	2188.63	2105.17	2105.01	1760.83
	Image entropy	5.04	4.39	4.72	4.50	4.77
	Time/s	9.54	9.40	9.39	9.42	9.50

**Table 3:** Comparison of evaluation index values of K-means, AP, DPC, GA-DPC, and FOA-DPC clustering algorithms.

Methods	Index	Experimental images					
		1	2	3	4	5	6
K-means	SEC	1351.75	1617.53	1561.35	1473.01	1305.70	1461.69
	Image entropy	1.62	1.75	2.18	2.16	1.88	2.06
	Time/s	0.50	0.71	0.45	0.41	0.99	0.46

AP	SEC	408.99	668.22	198.23	148.84	640.34	291.53
	Image entropy	1.23	1.31	1.47	1.16	1.40	1.35
	Time/s	0.29	0.28	0.21	0.22	0.25	0.21

DPC	SEC	2005.61	2177.19	1998.54	1904.61	1998.03	1961.34
	Image entropy	3.10	3.06	3.33	3.15	2.96	3.35
	Time/s	0.77	1.51	0.74	0.67	1.62	0.63

GA-DPC	SEC	2267.98	2059.73	2234.07	2129.66	2046.04	2252.31
	Image entropy	4.09	4.18	4.33	4.24	4.07	4.44
	Time/s	80.02	124.41	73.03	69.40	121.51	72.89

FOA-DPC	SEC	2364.77	2161.95	2059.37	2213.34	2157.90	2311.20
	Image entropy	4.36	4.54	4.56	4.51	4.39	4.72
	Time/s	44.06	72.49	40.69	40.02	69.72	43.60

## Data Availability

The data used to support the findings of this study are available from the corresponding author upon request.
